# Human Primary Macrophages Derived *In Vitro* from Circulating Monocytes Comprise Adherent and Non-Adherent Subsets with Differential Expression of Siglec-1 and CD4 and Permissiveness to HIV-1 Infection

**DOI:** 10.3389/fimmu.2017.01352

**Published:** 2017-10-23

**Authors:** Ousman Jobe, Jiae Kim, Eric Tycksen, Sayali Onkar, Nelson L. Michael, Carl R. Alving, Mangala Rao

**Affiliations:** ^1^U.S. Military HIV Research Program, Henry M. Jackson Foundation for the Advancement of Military Medicine, Bethesda, MD, United States; ^2^Laboratory of Adjuvant and Antigen Research, U.S. Military HIV Research Program, Walter Reed Army Institute of Research, Silver Spring, MD, United States; ^3^Genome Technology Access Center, Department of Genetics, Washington University in St. Louis, St. Louis, MO, United States; ^4^Laboratory of Molecular Virology and Pathogenesis, Host Genetics Section, U.S. Military HIV Research Program, Walter Reed Army Institute of Research, Silver Spring, MD, United States

**Keywords:** monocyte-derived macrophages, human immunodeficiency virus type 1, Siglec-1, CD4, RNA-Seq, restriction factors

## Abstract

Macrophages are a major target for human immunodeficiency virus type 1 (HIV-1) infection. However, macrophages are largely heterogeneous and may exhibit differences in permissiveness to HIV-1 infection. This study highlights the interplay of macrophage heterogeneity in HIV-1 pathogenesis. We show that monocyte-derived macrophages (MDMs) could be divided into two distinct subsets: CD14^+^Siglec-1^hi^CD4^+^ (non-adherent MDM) and CD14^+^Siglec-1^Lo^CD4^−^ (adherent MDM). The CD14^+^Siglec-1^hi^CD4^+^MDM subset represented the smaller proportion in the macrophage pool, and varied among different donors. Fractionation and subsequent exposure of the two MDM subsets to HIV-1 revealed opposite outcomes in terms of HIV-1 capture and infection. Although the CD14^+^Siglec-1^hi^CD4^+^MDM captured significantly more HIV-1, infection was significantly higher in the CD14^+^Siglec-1^Lo^CD4^−^MDM subset. Thus, CD14^+^Siglec-1^hi^CD4^+^MDM were less permissive to infection. Depletion of CD14^+^Siglec-1^hi^CD4^+^MDM or a decrease in their percentage, resulted in increased infection of MDM, suggestive of a capacity of these cells to capture and sequester HIV-1 in an environment that hinders its infectivity. Increased expression of innate restriction factors and cytokine genes were observed in the non-adherent CD14^+^Siglec-1^hi^CD4^+^MDM, both before and after HIV-1 infection, compared to the adherent CD14^+^Siglec-1^Lo^CD4^−^MDM. We speculate that the differential expression of gene expression profiles in the two macrophage subsets may provide an explanation for the differences observed in HIV-1 infectivity.

## Introduction

Macrophages are important targets of human immunodeficiency virus type 1 (HIV-1) infection (1–4) and may represent specialized viral reservoirs, with the ability to store HIV-1 particles in intracellular compartments (5, 6). It has been reported that infectious HIV-1 within macrophages are protected from neutralizing antibodies (7), further complicating HIV-1 eradication. Due to their dissemination over different tissues and their capacity to infiltrate virtually all organs including the brain, macrophages could likely contribute to the spread of HIV-1 and HIV-related pathologies, including immune dysfunction, persistent hyperimmune activation, and the onset of opportunistic infections (4, 8, 9).

In humans, macrophages arise from circulating or resident monocytes which are largely present in the blood, spleen and bone marrow. Circulating monocytes exhibit heterogeneity and are classified into classical monocytes, intermediate, and non-classical monocytes. Although earlier reports defined circulating monocytes as the precursors of tissue macrophages, recent studies have shown that tissue macrophages with self-renewal properties could arise from yolk sac, liver, and bone-marrow independent of monocyte precursors (10–12). Therefore, circulating monocytes represent only one source of tissue resident macrophages. The necessity of the circulating monocytes to repopulate macrophages in certain tissues versus the ability of macrophages to self renew in other tissues independent of circulating monocytes (13), highlights the complexity of tissue resident macrophage populations.

In human lungs, macrophage heterogeneity in the bronchoalveolar space is reflected by the presence of small and large alveolar macrophages, with small alveolar macrophages being more susceptible to HIV-1 infection than large alveolar macrophages (14). In the lungs of rhesus macaques, macrophage heterogeneity is exemplified by the presence of multiple macrophage populations including alveolar and interstitial macrophages (15), with interstitial macrophages being more permissive to simian immunodeficiency virus than alveolar macrophages (16).

Until recently, CD4 and chemokine receptors were the major cellular molecules associated with HIV-1 infection. However, recent studies have revealed the involvement of sialic acid-binding immunoglobulin-like lectin-1 (Siglec-1, CD169) in HIV-1 infection of myeloid cells. Siglec-1 is an interferon-inducible member of the I-type lectin receptor family found on the surface of dendritic cells and macrophages. *In vivo*, expression of Siglec-1 on myeloid cells is upregulated by immune activation, and these cells have been shown to accumulate in the CD4^+^ T cells-enriched lymphocyte tissues (17). Siglec-1 on dendritic cells captures HIV-1 by binding to sialyllactose-containing gangliosides exposed on HIV-1 membranes (18, 19). Siglec-1 also facilitates HIV-1 infection of macrophages *via* its interaction with sialic acid on gp120 (20, 21). It was recently reported that Siglec-1 mediated the accumulation of HIV-1 into virus-containing compartments of macrophages and also mediated the transinfection of autologous T cells (22).

In this study, using an *in vitro* infection system, we identified two distinct macrophage subsets, CD14^+^Siglec-1^hi^CD4^+^CD163^+^MDM and CD14^+^Siglec-1^Lo^CD4^−^CD163^−^MDM. We characterized their permissiveness to HIV-1 infection and their gene expression profiles in response to HIV-1. Our data revealed distinct differences in HIV-1 infectivity and anti-HIV-1 gene expression between the two-macrophage subsets. These results could have implications in the role of macrophages in HIV-1 pathogenesis.

## Materials and Methods

### Antibodies

The following human monoclonal antibodies (mAbs) anti-CD11b PE (clone ICRF44), CD11b FITC (clone ICRF44), CD14 APC (clone M5E2), CD14 PerCP (clone MoP9), CD163 FITC (clone GHI/61), CD4 PE (clone RPA-T4), CD3 PerCP (clone SK7), CD195 FITC (2D7/CCR5), and 7-amino actinomycin D (7-AAD) were obtained from BD Pharmingen. Anti-CD169 APC (clone 7-239) was obtained from BioLegend. Anti-p24-FITC and anti-p24-RD1 were purchased from Beckman Coulter.

### Media and Reagents

Media components and reagents were obtained as follows: RPMI-1640 (BioWhittaker), l-glutamine and penicillin/streptomycin (Quality Biologicals Inc.), Accutase (eBiosciences), recombinant human M-CSF (PeproTech), polybrene, bovine serum albumin (BSA), PKH-67, and PKH-26 (Sigma-Aldrich), and fetal bovine serum (Gemini Bio Products). Fixation and permeabilization buffers (Reagents A and B) were from Caltag.

Monocyte media consisted of RPMI-1640 supplemented with 10% heat-inactivated FBS, 1% l-glutamine, and 1% penicillin/streptomycin. M-CSF media (monocyte media supplemented with 50 ng/ml M-CSF) was used for differentiating the monocytes into macrophages. For infecting the macrophages, M-CSF media containing 2 µg/ml polybrene (Infection media) was used.

### Virus Purification

HIV-1 primary subtype B viruses (US-1, BaL, and JRFL) were grown in peripheral blood mononuclear cells (PBMCs) from stocks obtained from Dr. Victoria Polonis (USMHRP). The primary viruses were purified as previously described (23). Infectivity and p24 concentration were determined before and after purification to ensure that infectivity was not lost during the purification procedure.

### Enrichment and *In Vitro* Culture of Monocytes

Peripheral blood mononuclear cells from healthy HIV-1 seronegative donors were isolated by Ficoll density gradient centrifugation under an internal review board-approved protocol, RV229/WRAIR number 1386. Monocytes were enriched from the PBMCs by plastic adherence in 24-well plates (Corning), and differentiated into monocyte-derived macrophages (MDM) in 1 ml M-CSF media, as previously described (21). MDM were used on day 5 postculture for flow cytometry. For HIV-1 infection, polybrene (2 µg/ml) was added to the MDM cultures during the last 30 min of the *in vitro* culture, before subsequent exposure to HIV-1.

### Fractionation of MDM

M-CSF-derived MDM cultures comprised two cell fractions—adherent and non-adherent. The non-adherent MDM were isolated from their adherent counterparts by repeated gentle washes with monocyte media. The non-adherent MDM were gently aspirated, and collected in 50 ml tubes. Accutase (500 µl) was added to the remaining adherent MDM, and the cultures were incubated at 37°C/5% CO_2_ for 20 min, to detach the cells (24). The detached MDM were transferred into 50 ml tubes, and washed with monocyte media. The viability of both adherent and non-adherent MDM was ≥ 98% as determined by trypan blue exclusion.

### Detection of Cell Surface Molecules

Unfractionated or fractionated MDM (adherent and non-adherent) were washed in cold FACS buffer (PBS-containing 0.5% BSA) and blocked in FACS buffer containing 10% normal goat serum. The cells were incubated for 20 min on ice with a cocktail containing 5–10 µg of the specific mAb or their corresponding isotype mAbs as controls. Cells were washed in cold FACS buffer and fixed in PBS-containing 2% paraformaldehyde. Cells were acquired on a FACSCalibur (BD Biosciences, San Jose, CA, USA). Data analyses were performed on the gated 7-AAD negative MDM (CD14^+^) using FlowJo 8.8.6 software (TreeStar Inc., Ashland, OR, USA).

### Cell Sorting of MDM

Unfractionated MDM were harvested, pooled, and incubated with a mAb cocktail (CD14 FITC, CD4 PE, CD3 PerCP, Siglec-1 APC) on ice for 20 min. Cells were washed, and the pellet was resuspended in cold FACS buffer. An aliquot of the stained cells was acquired on a FACSCalibur before cell sorting. The remaining stained cells were sorted on an LSRII (BD Biosciences, San Jose, CA, USA). Data analyses were performed on the gated CD14^+^MDM, using FlowJo 8.8.6 software (TreeStar Inc., Ashland, OR, USA). The following gating strategy was used: Singlets were identified and gated by their forward scatter height (FSC-H) and area (FSC-A) characteristics. The live cells within the gated singlets were identified and gated. MDM in the gated live population were identified by their CD14^+^CD3^−^ characteristics. The defined CD14^+^CD3^−^ cells were gated and further defined into a Siglec-1 versus CD4 dot plot. The cells segregated into two populations, Siglec-1^hi^CD4^+^MDM and Siglec-1^Lo^CD4^−^MDM, which were subsequently collected into two separate tubes (Figure S1 in Supplementary Material).

### HIV-1 Capture and Replication in Fractionated MDM

In our previous study (21), the MDM were infected by spinoculation. In our current study, HIV-1 was gently mixed with the MDM cultures, with minimal perturbation, and incubated for the specified time-periods. The rationale for this modification was necessitated by our goal to maintain the MDM as non-adherent and adherent subsets. Although higher infectivity is achieved with spinoculation, this mechanical procedure would affix all the MDM to the plate, rendering it difficult to separate the non-adherent MDM from their adherent counterparts. Equal numbers (3–5 × 10^5^) of fractionated adherent MDM (Siglec-1^Lo^CD4^−^) and non-adherent MDM (Siglec-1^hi^CD4^+^) were each resuspended in 100 µl of infection media, transferred to 5 ml polystyrene tubes (Falcon), and incubated at 37°C/5% CO_2_ for 30 min followed by the addition of purified HIV-1 (1–5 ng p24) for an additional 3 h. Unadsorbed virus was removed following multiple washes (5×) with 1× PBS. Cells were lysed for determination of virus capture, and the lysates were evaluated for the presence of gag RNA by qRT-PCR. For evaluation of virus replication, the cell pellets were resuspended in 1 ml of infection media, transferred into 24-well flat-bottom plates, and incubated at 37°C/5% CO_2_. Culture supernatants and cells were harvested on day 4 postinfection. The presence of p24, indicative of HIV-1 infection, was determined in the two MDM subsets by flow cytomery (intracellular p24), and in the culture supernatants by ELISA (extracellular p24).

### HIV-1 Infection of Unfractionated and Siglec-1^hi^CD4^+^ Depleted MDM

Replicate wells of unfractionated MDM and Siglec-1^hi^CD4^+^(non-adherent)-depleted MDM were incubated in 1 ml of Infection media for 30 min at 37°C/5% CO_2_. Purified HIV-1 (1–5 ng p24) was added to each well and the cultures were incubated at 37°C/5% CO_2_. Cultures were harvested on day 4 postinfection and analyzed for the presence of intracellular p24 by flow cytometry.

### Coculture Assays

Unfractionated MDM were exposed to HIV-1 or media for 1 h. Cells were fractionated into non-adherent (Siglec-1^hi^CD4^+^) and adherent (Siglec-1^Lo^CD4^−^) MDM, and washed to remove unadsorbed virus. Siglec-1^hi^CD4^+^MDM were pooled and counted. Duplicate wells of Siglec-1^Lo^CD4^−^MDM were counted as an indicator for the number of Siglec-1^Lo^CD4^−^MDM in each well. Equal numbers of HIV-1-exposed non-adherent Siglec-1^hi^CD4^+^MDM were cocultured with HIV-1 naive PKH-67-labeled adherent Siglec-1^Lo^CD4^−^MDM. HIV-1 naive Siglec-1^hi^CD4^+^MDM were also cocultured with HIV-1 exposed PKH-67-labeled Siglec-1^Lo^CD4^−^MDM. Independent cultures of HIV-1-exposed non-adherent Siglec-1^hi^CD4^+^MDM and HIV-1-exposed PKH-26-labeled adherent Siglec-1^Lo^CD4^−^MDM were setup in parallel. The cultures were incubated for 2–3 days at 37°C/5% CO_2_, harvested, and analyzed for HIV-1 infectivity by flow cytometry.

### qRT-PCR

RNA was extracted from HIV-1-infected MDM subsets and the presence of gag RNA was determined by qRT-PCR as previously described (21, 25). Briefly, RNA was extracted from MDM using the RNeasy Mini Kit and Qiashredder (Qiagen) and the RNA was eluted in RNase free water. The qRT-PCR reactions were performed using the TaqMan RNA-to-Ct master mix (Applied Biosystems) and Viia7 (Applied Biosystems). Reactions (50 µl) were performed in the presence of the master mix, 0.2 µM each of Gag forward and reverse primers, Gag probe and 1× human GAPDH VIC-TAMRA (Applied Biosystems). Cycling parameters were 48°C for 20 min, 95°C for 10 min; then 45 cycles at 95°C for 15 s, and 59°C for 1 min. Delta Ct values were calculated to normalize the HIV-1 gag RNA signal as a function of the GAPDH/cellular RNA signal.

### Detection of Intracellular and Extracellular HIV-1 p24 Antigen

Staining for detection of intracellular p24 in HIV-1 infected MDM was carried out as previously described (23). Data analyses were performed using FlowJo 8.8.6 software (TreeStar Inc., Ashland, OR, USA). The concentration of extracellular HIV gag p24 in the culture supernatants was determined using a HIV-1 p24 Antigen Capture Assay kit (ABL).

### RNA-Seq Analysis of Cellular Genes

RNA was isolated from uninfected and HIV-1 infected MDM using the RNeasy Mini Kit (Qiagen) and the concentration was determined using Nanodrop 2000 (Thermo Scientific). The eluted RNA had a 260/280 of greater than 1.8. The samples were analyzed for quality on an Agilent BioAnalyzer and all samples had a RNA Integrity Number (RIN) value of greater than 9.5. Samples were then prepared for sequencing with the Clontech SMARTer system, indexed, pooled, and sequenced as a single 1 × 50 bp lane on an Illumina HiSeq 3000. RNA-seq reads were demultiplexed and aligned to the Ensembl release 76 top-level assembly with STAR version 2.0.4b. Gene counts were derived from the number of uniquely aligned unambiguous reads by Subread:featureCount version 1.4.5. Sequencing performance was assessed for total number of aligned reads, total number of uniquely aligned reads, and genes detected. The ribosomal fraction (Figure S2 in Supplementary Material), known junction saturation (Figure S3 in Supplementary Material), and read distribution over known gene models (Figure S4 in Supplementary Material) were quantified with RSeQC version 2.3.

All gene counts were then imported into the R/Bioconductor package EdgeR and TMM normalization size factors were calculated to adjust for samples for differences in library size. Ribosomal genes and genes not expressed in any sample greater than one count-per-million were excluded from further analysis. The TMM size factors and the matrix of counts were then imported into R/Bioconductor package Limma. Performance of the samples was assessed with a Spearman correlation matrix. Sample outliers with confounding levels of variance found in the correlation plot were removed from further analysis (Figure S5 in Supplementary Material). Weighted likelihoods based on the observed mean-variance relationship of every gene and sample were then calculated for all samples with the voom WithQualityWeights function and gene performance was assessed with plots of residual standard deviation of every gene to their average log-count with a robustly fitted trend line of the residuals (Figure S6 in Supplementary Material). Generalized linear models were then created to test for gene level differential expression and the results were filtered for only those genes with *p*-values ≤0.05 and log 2 fold-changes greater than an absolute value of 2.

For each contrast extracted with Limma, global perturbations in known Gene Ontology (GO) terms and KEGG pathways were detected using the R/Bioconductor packages GAGE to test for changes in expression of the reported log 2 fold-changes reported by Limma in each term versus the background log 2 fold-changes of all genes found outside the respective term. The R/Bioconductor package heatmap3 was used to display heatmaps of genes across samples for each GO terms with a *p*-value ≤0.05. The R/Bioconductor package Pathview was then used to generate annotated pathway maps on perturbed KEGG signaling and metabolism pathways. The logFC values reported in column B in Table S1 in Supplementary Material are the fold-changes as reported by Limma’s weighted generalized linear model likelihood ratio test for the contrast of adherent and non-adherent MDM’s (26–29).

### Accession Number

The accession number for the raw and processed files for the RNA-seq reported in this article is GEO: GSE103666.

### Statistical Analysis

Differences were compared using the Mann-Whitney test (Graphpad Prism 5, Version 5.0c). A *p*-value of ≤0.05 was considered statistically significant. Statistical analyses for the RNA-Seq data were performed as mentioned above in the RNA-Seq analysis of cellular genes.

## Results

### M-CSF-Derived MDM Comprised Two Subsets-Siglec-1^hi^CD4^+^MDM and Siglec-1^Lo^CD4^−^MDM

**Figure 1:**
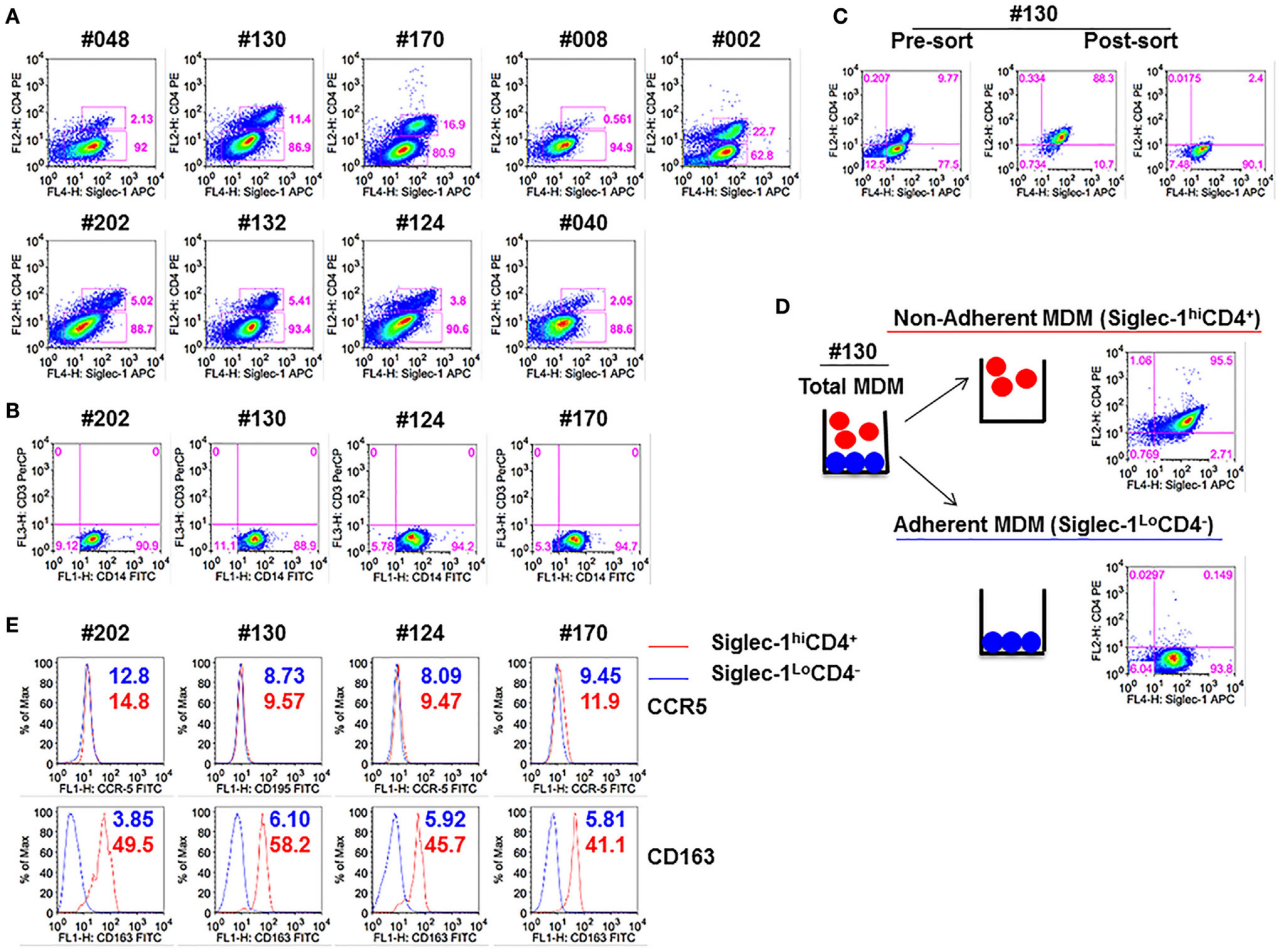
Monocyte-derived macrophage (MDM) segregate into two distinct subsets: Non-adherent Siglec-1^hi^CD4^+^MDM and adherent Siglec-1^Lo^CD4^−^MDM. Triplicate wells of primary human monocytes from nine human immunodeficiency virus (HIV)-seronegative donors (#048, #130, #170, #008, #002, #202, #132, #124, and #040) were differentiated into MDM following *in vitro* culture in M-CSF media for 5 days. Cultures were harvested, stained, and analyzed by flow cytometry. **(A)** Plots show percentage of Siglec-1^hi^CD4^+^MDM and Siglec-1^Lo^CD4^−^MDM within the gated CD14^+^ cells. A representative plot of one of the two independent experiments is shown. **(B)** Plots show that gated CD14^+^ cells are not contaminated with CD3^+^ T cells. **(C,D)** *In vitro* cultures of M-CSF-derived MDM contain adherent and non-adherent MDM. **(C)** Adherent and non-adherent MDM were pooled, stained, and the gated CD14^+^ cells were sorted into Siglec-1^hi^CD4^+^MDM and Siglec-1^Lo^CD4^−^MDM. **(D)** Non-adherent MDM were separated from adherent MDM following repeated washing with media. Adherent MDM were detached with Accutase. Both fractions were washed, stained, and the gated CD14^+^ cells were analyzed separately for the expression of Siglec-1 and CD4. Plots show that non-adherent fraction represented the Siglec-1^hi^CD4^+^MDM subset, whereas the adherent fraction comprised the Siglec-1^Lo^CD4^−^MDM subset. **(E)** Histograms show the expression of CCR5 and CD163 on the gated Siglec-1^Lo^CD4^−^MDM (blue) and Siglec-1^hi^CD4^+^MDM (red) subsets. Values in the histograms denote the mean fluorescent intensity (MFI) of the specific receptors for each of the subsets. Each experiment was done twice in triplicate and the data from one of the two experiments are shown.

We have previously demonstrated that blocking Siglec-1 receptor on MDM resulted in 90–95% inhibition of HIV-1 infection, whereas blocking CD4 receptor inhibited infection in 50–55% of MDM (21). Thus, we investigated the possible existence of subsets of MDM that coexpressed varying amounts of Siglec-1 and CD4. Both adherent and non-adherent cells were evident in the M-CSF-derived MDM cultures. Flow cytometric analysis of the combined adherent and non-adherent fractions showed that M-CSF-derived MDM, irrespective of the donor, segregated into two distinct subsets: Siglec-1^hi^CD4^+^ and Siglec-1^Lo^CD4^−^ (Figure 1A). Two notable observations were made. First, within the MDM cultures in all nine donors, Siglec-1^hi^CD4^+^MDM comprised a lower proportion of cells (0.6–22.7%) compared to Siglec-1^Lo^CD4^−^MDM (62.8–94.9%). Second, the proportion of Siglec-1^hi^CD4^+^MDM varied among donors (Table 1). Both MDM subsets expressed similar frequencies of CD14, and were negative for CD3, implying the absence of T-cell contamination (Figure 1B).

**Table 1:** Mean fluorescence intensity (MFI) of Siglec-1 for Siglec-1^hi^CD4^+^MDM and Siglec-1^Lo^CD4^−^MDM from various donors.

Donor	Siglec-1^hi^CD4^+^MDM	Siglec-1^Lo^CD4^−^MDM
#048	46.7	29.1
#130	188.0	29.5
#170	142.0	66.3
#008	88.3	19.0
#002	80.5	66.3
#202	167.0	14.6
#132	175.0	41.6
#124	143.0	20.4
#040	66.5	17.1

Since the main goal of this study was to individually evaluate the two different MDM subsets for HIV-1 infection, we utilized two different methods (cell sorting and cell fractionation) to separate the two MDM subsets. For cell sorting, the MDM were stained with a mAb cocktail and subjected to sorting by flow cytometry (Figure 1C). MDM were sorted into the two subsets (Siglec-1^hi^CD4^+^ and Siglec-1^Lo^CD4^−^) with 89–90% purity. The second method employed was cell fractionation (Figure 1D). The fractionation procedure was based on the differential adherence characteristics of the two different subsets of MDM. Although, both procedures (Figures 1C,D) yielded highly enriched and pure subsets, the fractionation procedure was simple, inexpensive, gentle, fast, and circumvented the possible effects of antibody exposure and subsequent mechanical cell sorting. With all donors, routine flow cytometric analyses of the fractionated MDM subsets consistently revealed that the non-adherent MDM represented the Siglec-1^hi^CD4^+^MDM, whereas the adherent MDM comprised the Siglec-1^Lo^CD4^−^MDM (data not shown). Therefore, we utilized the fractionation procedure for the experiments described in this study. Evaluation of HIV-1 coreceptor (CCR5) revealed that the expression of CCR5 was similar on both subsets, whereas the scavenger receptor (CD163) was only coexpressed on Siglec-1^hi^CD4^+^MDM (Figure 1E). These data reveal the existence of two subsets of M-CSF-derived MDM; a small subset of Siglec-1^hi^CD4^+^CD163^+^MDM (non-adherent), with a relatively high expression of molecules that are associated with HIV-1 infection, and a more prominent subset of Siglec-1^Lo^CD4^−^CD163^−^MDM (adherent), with a relatively lower expression of Siglec-1, and devoid of CD4 and CD163.

### Siglec-1^hi^CD4^+^MDM Dampens the Degree of HIV-1 Infectivity

**Figure 2:**
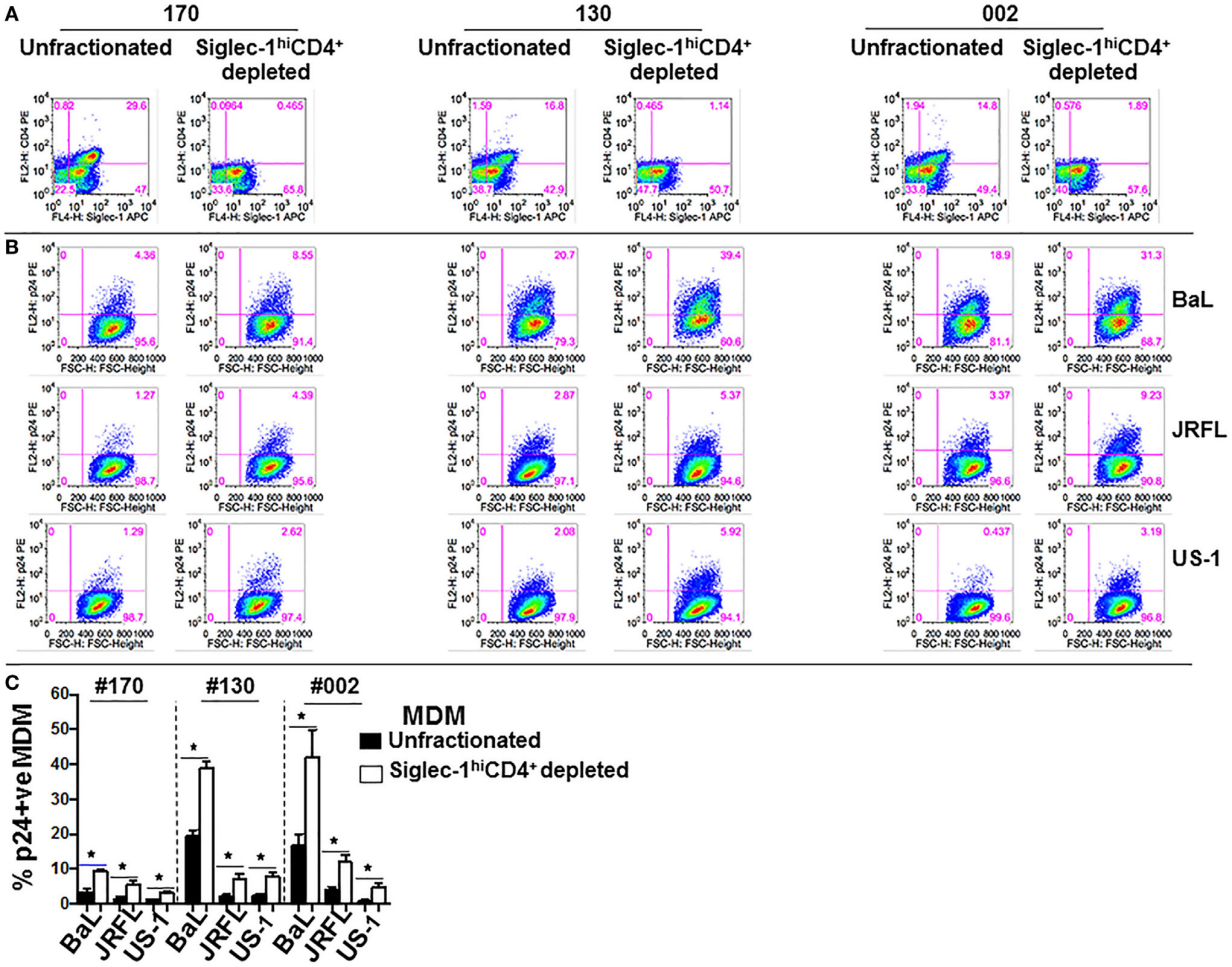
Presence of Siglec-1^hi^CD4^+^MDM dampens the degree of human immunodeficiency virus type 1 (HIV-1) infection. Monocytes from three donors were differentiated into monocyte-derived macrophage (MDM) with M-CSF media. **(A)** Panels show plots of unfractionated MDM and their Siglec-1^hi^CD4^+^MDM-depleted counterparts. **(B)** Unfractionated MDM and Siglec-1^hi^CD4^+^MDM-depleted cultures were infected with purified HIV-1 (BaL, JRFL, or US-1). Cells were harvested on day 3 postinfection, stained, and analyzed by flow cytometry. Panels show plots of HIV-1 infection in the unfractionated MDM cultures and in their Siglec-1^hi^CD4^+^MDM-depleted counterparts. Values in the upper right quadrant(s) represent the percentage of infected MDM. Data represent one of the triplicate wells from one of two independent experiments. **(C)** Bar graph is derived from the experiment performed in Figure 2B and includes the data from the triplicate wells of two independent experiments and shows the% p24^+^MDM (mean ± SD) in unfractionated MDM (filled bars) or in the Siglec-1^hi^CD4^+^MDM-depleted MDM (open bars) following infection with HIV-1. Significance is indicated by *p ≤ 0.05.

The presence of relatively high levels of molecules that are associated with HIV-1 infection on the M-CSF-derived MDM subset led us to hypothesize that the non-adherent Siglec-1^hi^CD4^+^MDM would be highly permissive to HIV-1 infection. To address this hypothesis, we evaluated the degree of HIV-1 infection in unfractionated and Siglec-1^hi^CD4^+^ depleted MDM. We utilized the fractionation procedure mentioned above in three donors (#170, #130, and #002), to remove non-adherent Siglec-1^hi^CD4^+^MDM from the adherent MDM population. The efficiency of the fractionation procedure to deplete Siglec-1^hi^CD4^+^MDM from the cultures was confirmed by flow cytometry (Figure 2A). Unfractionated MDM and Siglec-1^hi^CD4^+^ depleted MDM from multiple donors were infected with three different HIV-1 subtype B purified viruses (BaL, JRFL, and US-1). The degree of infectivity was variable among the donors with each of the viruses. Surprisingly, with all the viruses and in all the donors, HIV-1 infection was consistently and significantly higher (*p* ≤ 0.05) in Siglec-1^hi^CD4^+^MDM-depleted cultures compared to unfractionated MDM cultures, (Figures 2B,C). Our data demonstrate that in an *in vitro* infection set-up, Siglec-1^hi^CD4^+^MDM dampened the effect on the degree of HIV-1 infection. This suggests that *in vitro*, the outcome of HIV-1 infection may be related to the percentage of Siglec-1^hi^CD4^+^MDM in the well.

### Siglec-1^hi^CD4^+^MDM Are Efficient at HIV-1 Capture but Less Permissive to Infection

**Figure 3:**
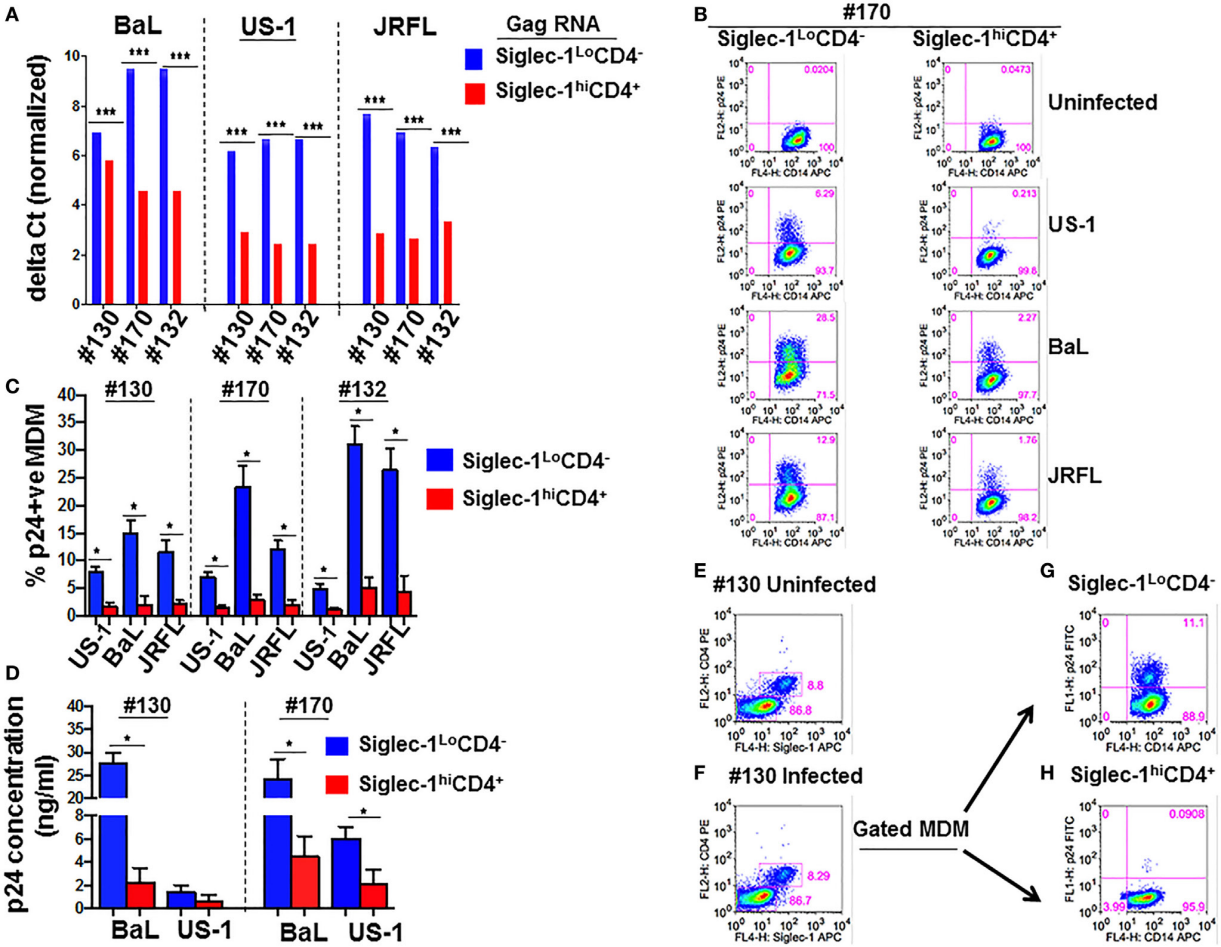
Human immunodeficiency virus type 1 (HIV-1) infection is lower in Siglec-1^hi^CD4^+^MDM despite higher virus capture. Equal numbers (3 × 10^5^ cells) of Siglec-1^hi^CD4^+^MDM and Siglec-1^Lo^CD4^−^MDM from three HIV-seronegative donors (#130, #170, #132) were incubated with HIV-1 (BaL, JRFL, or US-1) for 3 h at 37°C/5% CO_2_. **(A)** Cell lysates were subjected to qRT-PCR to detect gag RNA transcripts, indicative of virus capture. Uninfected cell lysates were negative for gag RNA transcripts (not shown). Bar graphs show the delta Ct (normalized) gag RNA in Siglec-1^hi^CD4^+^MDM (red bars) and Siglec-1^Lo^CD4^−^MDM (blue bars) for each donor and with each virus. Data are representative of three independent experiments and show the mean values (***p ≤ 0.001). **(B,C)** Siglec-1^hi^CD4^+^MDM and Siglec-1^Lo^CD4^−^MDM were infected with HIV-1. Cultures were harvested on day 4 postinfection, washed, stained, and analyzed for the presence of intracellular p24 by flow cytometry. **(B)** Panels show plots of HIV-1 infection in gated Siglec-1^hi^CD4^+^MDM and Siglec-1^Lo^CD4^−^MDM from a representative donor. **(C)** Bar graph shows % p24^+^MDM of triplicate wells of three independent experiments (mean ± SD; *p ≤ 0.05) in Siglec-1^Lo^CD4^−^MDM (blue bars) and in the Siglec-1^hi^CD4^+^MDM (red bars) following infection with US-1, BaL, or JRFL. **(D)** Supernatants from cultures of HIV-1-infected Siglec-1^hi^CD4^+^MDM and Siglec-1^Lo^CD4^−^MDM were harvested on day 4 postinfection, and analyzed by ELISA for the presence of extracellular p24. Bar graphs show the p24 concentration of triplicate wells of two independent experiments (mean ± SD; *p ≤ 0.05) in the supernatants of HIV-1-infected Siglec-1^Lo^CD4^−^MDM (blue bars) and Siglec-1^hi^CD4^+^MDM (red bars) for each donor and with each virus. **(E,F)** Siglec-1^Lo^CD4^−^MDM (#130) were left uninfected, or **(F)** were infected with HIV-1. Plots show the presence of Siglec-1^hi^CD4^+^MDM both in **(E)** the uninfected Siglec-1^Lo^CD4^−^MDM cultures and in **(F)** their HIV-1-infected Siglec-1^Lo^CD4^−^MDM cultures. **(G,H)** The HIV-1-infected monocyte-derived macrophage (MDM) described in **(F)** were analyzed for localization of HIV-1 on the basis of their Siglec-1^Lo^CD4^−^MDM versus Siglec-1^hi^CD4^+^MDM phenotype. Panels show that the presence of HIV-1 infection only in **(G)** gated Siglec-1^Lo^CD4^−^MDM and not in the **(H)** gated Siglec-1^hi^CD4^+^MDM. Values in the upper right quadrant(s) represent the percentage of infected MDM. A representative plot of three independent experiments is shown.

We sought to further investigate the interaction of the two MDM subsets with HIV-1. In this regard, we evaluated if the two subsets exhibited differences in their ability to capture virus, and/or support infection. Equal numbers of fractionated Siglec-1^Lo^CD4^−^MDM and Siglec-1^hi^CD4^+^MDM from different donors were exposed to three different subtype B HIV-1 (BaL, US-1, JRFL). First, we evaluated HIV-1 capture by the two subsets (Figure 3A). This was determined by the presence of gag RNA using qRT-PCR. The cells were harvested at 3 h postinfection. RNA was isolated from cell lysates and qRT-PCR was performed. The data are plotted as ΔCt values. A low ΔCt value represents a higher amount of viral RNA. As shown in Figure 3A, HIV-1 capture of all three viruses was significantly higher in the Siglec-1^hi^CD4^+^MDM (lower Ct value), compared to Siglec-1^Lo^CD4^−^MDM (higher Ct value) in all three donors. Next, we evaluated HIV-1 infection at day 4 postinfection. HIV-1 infection was determined by the presence of intracellular p24 using flow cytometry (Figures 3B,C), as well as the presence of extracellular p24 in the supernatants using ELISA (Figure 3D). Interestingly, in all the donors and with all three viruses, the percentage of p24 positive MDM was significantly higher in the Siglec-1^Lo^CD4^−^MDM compared to the Siglec-1^hi^CD4^+^MDM subset (Figures 3B,C). Parallel determination of extracellular p24 in the culture supernatants also revealed significantly higher levels in Siglec-1^Lo^CD4^−^MDM cultures, than in the Siglec-1^hi^CD4^+^MDM cultures (Figure 3D). Collectively, these results suggest that although Siglec-1^Lo^CD4^−^MDM are less efficient at HIV-1 capture than Siglec-1^hi^CD4^+^MDM, they are more permissive to HIV-1 infection.

Although the fractionated Siglec-1^Lo^CD4^−^MDM were initially devoid of Siglec-1^hi^CD4^+^MDM and were cultured as a relatively pure subset, we detected the presence of Siglec-1^hi^CD4^+^MDM at day 4 postinfection in the uninfected controls (Figure 3E), as well as in the infected Siglec-1^Lo^CD4^−^MDM cultures (Figure 3F). This is indicative of macrophage plasticity, and suggests that Siglec-1^Lo^CD4^−^MDM are the precursors of Siglec-1^hi^CD4^+^MDM. Notably, HIV-1 infection was mainly evidenced in the gated Siglec-1^Lo^CD4^−^MDM (Figure 3G), but not in the Siglec-1^hi^CD4^+^MDM (Figure 3H).

### Siglec-1^hi^CD4^+^MDM Although Poorly Permissive Do Transfer HIV-1 to Siglec-1^Lo^CD4^−^MDM

**Figure 4:**
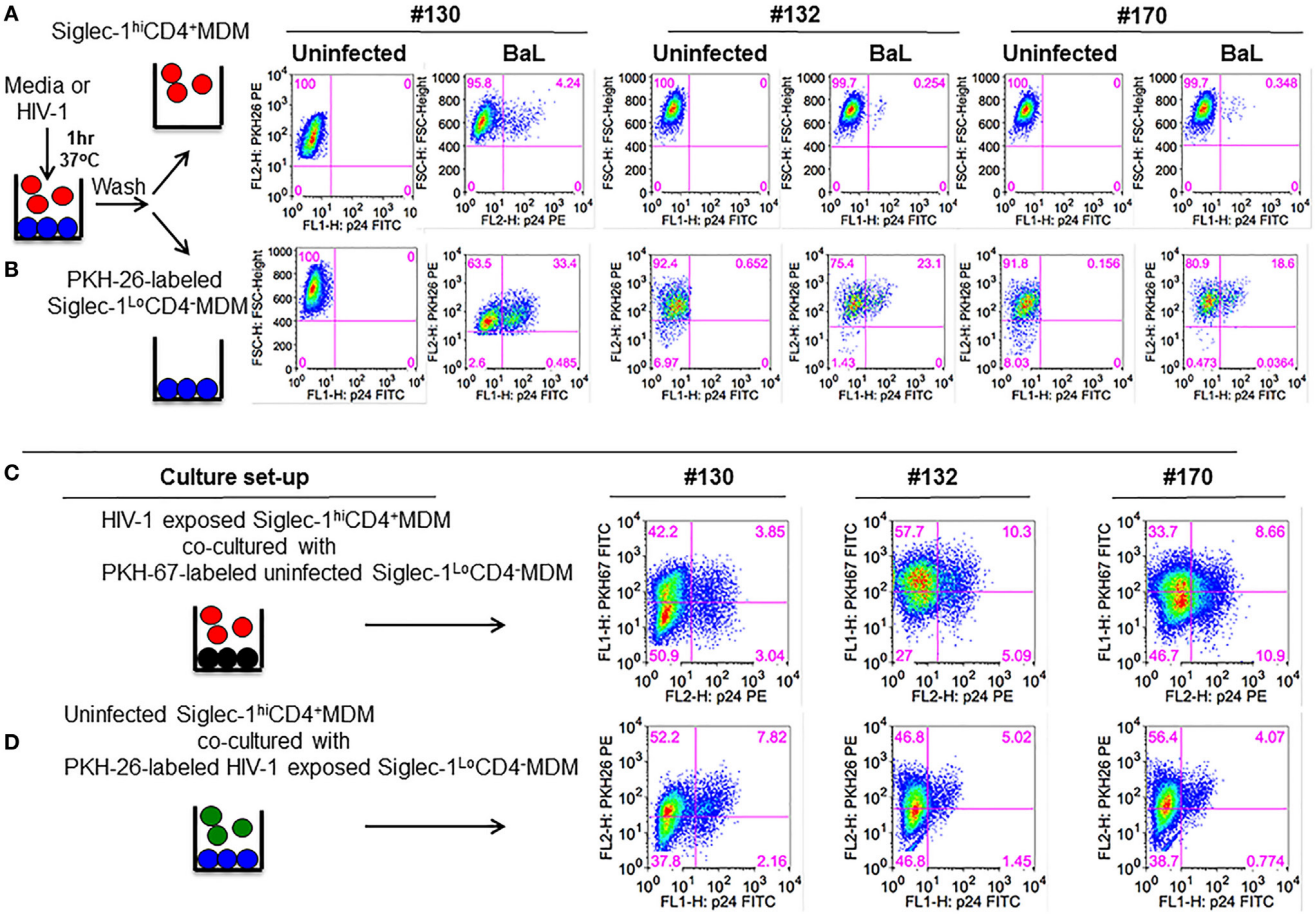
Siglec-1^hi^CD4^+^MDM transfer human immunodeficiency virus type 1 (HIV-1) to Siglec-1^Lo^CD4^−^MDM. **(A)** M-CSF-derived monocyte-derived macrophage (MDM) from three donors were incubated with media or with HIV-1 (BaL) for 1 h at 37°C/5% CO_2_. Cells were washed and fractionated into Siglec-1^hi^CD4^+^MDM and Siglec-1^Lo^CD4^−^MDM. The infected Siglec-1^Lo^CD4^−^MDM were subsequently labeled with PKH-26. Both subsets were cultured at 37°C/5% CO_2_. **(A)** Plots show the percentage of infected cells at 3 days postculture in the infected Siglec-1^hi^CD4^+^MDM and **(B)** the infected Siglec-1^Lo^CD4^−^MDM. **(C)** HIV-1 exposed Siglec-1^hi^CD4^+^MDM were cocultured with PKH-67-labeled uninfected Siglec-1^Lo^CD4^−^MDM. Plots show evidence of HIV-1 infection in both the Siglec-1^hi^CD4^+^MDM and the PKH-67-labeled Siglec-1^Lo^CD4^−^MDM. **(D)** In parallel, PKH-26-labeled HIV-1-exposed Siglec-1^Lo^CD4^−^MDM were cocultured with uninfected Siglec-1^hi^CD4^+^MDM. Plots show evidence of HIV-1 infection mostly in the PKH-26-labeled Siglec-1^Lo^CD4^−^MDM.

Despite increased HIV-1 capture (Figure 3A), virus replication was restricted in Siglec-1^hi^CD4^+^MDM (Figures 3B–D). It may be possible that Siglec-1^hi^CD4^+^MDM only captured and sequestered HIV-1. However, it is equally plausible that Siglec-1^hi^CD4^+^MDM facilitated transfer of captured virions to other cells. To evaluate this, we exposed unfractionated M-CSF-derived MDM to BaL for 1 h. The HIV-1-exposed MDM were fractionated into non-adherent and adherent populations. Phenotypic analysis of aliquots of the non-adherent and adherent fractions confirmed that they were Siglec-1^hi^CD4^+^MDM and Siglec-1^Lo^CD4^−^MDM, respectively (data not shown). The HIV-1-exposed Siglec-1^Lo^CD4^−^MDM were labeled with PKH-26, a cell membrane-labeling dye. The HIV-1-exposed MDM subsets were cultured independently, and served as infection controls (Figure 4A,B). Although HIV-1 infection was detected in both subsets, it was higher in the Siglec-1^Lo^CD4^−^MDM compared to the Siglec-1^hi^CD4^+^MDM in all three donors (Figure 4A,B). These data further highlighted that the Siglec-1^hi^CD4^+^MDM subset were less permissive to HIV-1 infection and are consistent with the results shown in Figures 3B–D.

To evaluate whether the HIV-1-exposed MDM subsets exhibited differences in their ability to transfer HIV-1, Siglec-1^hi^CD4^+^MDM from the three donors that were exposed to BaL were cocultured for 3 days with uninfected Siglec-1^Lo^CD4^−^MDM that were labeled with PKH-67 to distinguish them from Siglec-1^hi^CD4^+^MDM or from Siglec-1^hi^CD4^+^MDM that may have down-regulated Siglec-1 and CD4 during infection. As shown in Figure 4C, HIV-1 infection (3.85, 10.3, and 8.66%, respectively) was evident in the PKH-67-labeled Siglec-1^Lo^CD4^−^MDM in all three donors. This indicated that the HIV-exposed Siglec-1^hi^CD4^+^MDM transferred virus to the uninfected PKH-67-labeled Siglec-1^Lo^CD4^−^MDM.

In parallel, uninfected Siglec-1^hi^CD4^+^MDM were cocultured with HIV-exposed Siglec-1^Lo^CD4^−^MDM that were labeled with PKH-26 (Figure 4D). The coculture resulted in decreased infection (Figures 4B,D), suggestive of an inherent ability of Siglec-1^hi^CD4^+^MDM to restrict HIV-1 infection. These results are consistent with the data shown in Figure 2. The HIV-1-infected cells were largely restricted within the HIV-exposed PKH-26-labeled Siglec-1^Lo^CD4^−^MDM.

### Differential Expression of HIV-1 Restriction Factors and Cytokine Genes in Siglec-1^hi^CD4^+^MDM and Siglec-1^Lo^CD4^−^MDM

**Figure 5:**
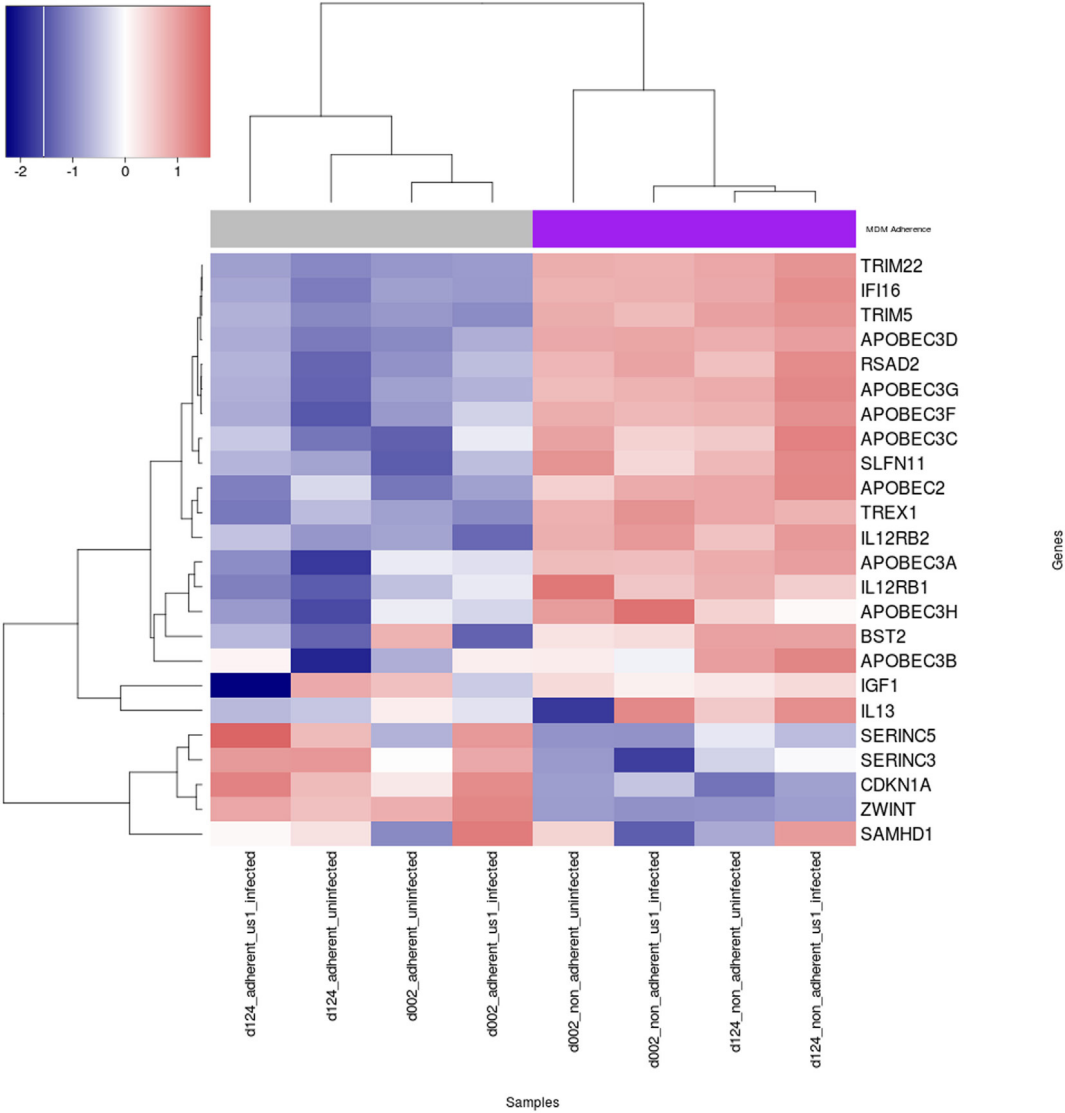
Heat map of expression of human immunodeficiency virus type 1 (HIV-1) restriction factors and cytokine genes in uninfected and HIV-1-infected Siglec-1^hi^CD4^+^MDM and Siglec-1^Lo^CD4^−^MDM. M-CSF-derived monocyte-derived macrophage (MDM) from two donors were fractionated into Siglec-1^hi^CD4^+^MDM and Siglec-1^Lo^CD4^−^MDM. The MDM subsets were incubated with media or with HIV-1 (BaL) for 3 h at 37°C/5% CO_2_. The expression levels of cellular restriction factors, insulin growth factor-1 (IGF-1), IL-13, and IL-12-related genes were analyzed by RNA-Seq. Relative expression levels of HIV-1 restriction genes are shown for uninfected and infected Siglec-1^hi^CD4^+^MDM and Siglec-1^Lo^CD4^−^MDM samples with a self-clustering heatmap generated by the R/CRAN package heatmap3. Samples and genes are clustered by their respective expression profiles and every row is rescaled to have a mean of zero and standard deviation of 0 in order to illustrate relative expression changes across samples on a scale similar to log 2 fold-change. Blue indicates low expression, and red indicates high expression.

The expression of cellular restriction factors and cytokine genes that affect HIV-1 infection in macrophages have been previously described (30–39). To gain more insight into the possible reasons for the differential permissivity to HIV-1 infection observed in our study, we examined M-CSF-derived MDM from three donors that were fractionated into Siglec-1^Lo^CD4^−^MDM and Siglec-1^hi^CD4^+^MDM, in the absence or presence of HIV-1 for 3 h. The expression levels of cellular restriction factors and cytokine-related genes were analyzed by RNA-Seq (Figure 5; Table S1 in Supplementary Material). One of the donors, #130 was inconsistent in the data compared to the other two donors (Figure S5 in Supplementary Material) and therefore the data from #130 was removed. Known genes for cellular restriction factors and cytokines that were considered significantly different in the two subsets, Siglec-1^Lo^CD4^−^MDM and Siglec-1^hi^CD4^+^MDM were chosen. Of these, the 23 genes selected were within the top 7,500 differentially expressed genes. We observed a trend of higher cellular restriction factor gene expression including viperin (RSAD2), SLFN11, IFI16, TREX1, APOBEC family, tetherin (BST2), TRIM5, and TRIM22 in the non-adherent Siglec-1^hi^CD4^+^MDM compared to the adherent Siglec-1^Lo^CD4^−^MDM. This pattern of differential expression was observed in the uninfected subsets and remained in the HIV-1 infected Siglec-1^Lo^CD4^−^MDM and Siglec-1^hi^CD4^+^MDM. Viperin (RSAD2), TRIM22, IFI16, and APOBEC3D were all within the top 200 differentially expressed genes. Surprisingly, the expression of SAMHD1 (40–42), SERINC5, and SERINC3 (43, 44), was higher in the HIV-1 permissive Siglec-1^Lo^CD4^−^MDM compared to the less permissive Siglec-1^hi^CD4^+^MDM, despite their innate antiviral properties. Cyclin-dependent kinases (CDK1, CDK2) which phosphorylate SAMHD1 and impair its HIV-restriction ability (45–47), showed a higher expression in Siglec-1^Lo^CD4^−^MDM compared to Siglec-1^hi^CD4^+^MDM. In addition to restriction factors, cytokines such as IL-12 and IL-13 also influence HIV-1 replication in MDM (32, 33, 48). Expression of the genes for the IL-12R and IL-13 were higher in the HIV-1-restrictive Siglec-1^hi^CD4^+^MDM. This subset also showed higher gene expression for insulin growth factor-1 (IGF-1), which has been previously demonstrated to be present in activated alveolar macrophages and to inhibit HIV-1 replication in cultured cord blood mononuclear cells as well as in chronically HIV-1 infected U937 cells (30, 49). These trends in gene expression profiles show that several factors that restrict HIV-1 infectivity were more highly expressed in the non-adherent Siglec-1^hi^CD4^+^MDM compared to the adherent Siglec-1^Lo^CD4^−^MDM and is suggestive of the restricted HIV-1 infection that we observed.

## Discussion

Macrophages are important targets for HIV-1 infection (50). In a recent study, it was shown that macrophages were capable of sustaining HIV-1 infection in the absence of T cells (4). In compartments such as the central nervous system, macrophages are the principal targets for HIV-1 (51). HIV-1-infected tissue-resident macrophages are largely resistant to the cytopathic effects of the virus. Unlike HIV-1-infected CD4^+^ T cells which are progressively depleted by mechanisms including apoptosis, infected macrophages are largely resistant to apoptosis (52). As a result, macrophages can harbor HIV-1 for extended periods (53–55), making them a major factor in the establishment of the viral reservoir (56). Macrophages are central to the innate immune response, and may be important in the control of opportunistic infections. It is therefore conceivable that perturbation of macrophage responses and functions as a result of HIV-1 infection may impair innate immune control and allow for the onset of opportunistic infections.

In the present study, MDM were used. However, macrophages are not all derived from circulating monocytes. Indeed, accumulating evidence suggests the presence of resident macrophages in several tissues that self-renew locally throughout adult life, independently of circulating monocytes (10, 11, 13, 57). Nonetheless, irrespective of the donors tested in our study, the MDM segregated into two distinct subsets; non-adherent and adherent. The two subsets exhibited a differential expression of cellular receptors associated with HIV-1 such as Siglec-1, CD4, and CD163. The non-adherent MDM were Siglec-1^hi^CD4^+^ and the adherent MDM were Siglec-1^Lo^CD4^−^. The non-adherent Siglec-1^hi^CD4^+^MDM comprised the smaller subset and varied among the donors. Although both subsets expressed comparable levels of CD14 and CCR5, only the non-adherent Siglec-1^hi^CD4^+^MDM expressed CD163. Interestingly, *in vitro* culture of fractionated adherent Siglec-1^Lo^CD4^−^MDM gave rise to non-adherent Siglec-1^hi^CD4^+^MDM, suggesting that Siglec-1^Lo^CD4^−^MDM may be the precursors of Siglec-1^hi^CD4^+^MDM. This highlights the plasticity of MDM and is in line with a previous observation that showed adherent interstitial lung macrophages as the precursors of non-adherent alveolar macrophages (15).

We found that significantly more HIV-1 was captured by the non-adherent Siglec-1^hi^CD4^+^MDM than their adherent Siglec-1^Lo^CD4^−^MDM counterparts. We and others have reported that Siglec-1 is an attachment molecule for HIV-1 (20, 21). Therefore, it is probable that the higher expression of HIV-1 associated cellular receptors on Siglec-1^hi^CD4^+^MDM may have resulted in a propensity for more efficient virus capture. Interestingly, although more HIV-1 was captured by the non-adherent Siglec-1^hi^CD4^+^MDM, infection was significantly restricted in these cells. Indeed, irrespective of donor, HIV-1 infection with BaL, US-1, or JRFL was consistently lower in the non-adherent Siglec-1^hi^CD4^+^MDM, than in the adherent Siglec-1^Lo^CD4^−^MDM.

Our observation that HIV-1 permissiveness was subset related has been reported in other cell types. In HIV-1-infected patients, evidence of HIV-1 infection is detected in only a very small number of monocytes (54). Characterization of monocyte subsets revealed that CD16^+^ monocytes were more permissive to HIV-1 than CD16^−^ monocytes (58, 59), demonstrating that CD16^+^ monocytes are the HIV-1 permissive monocyte subset. In the lungs of HIV-1 infected subjects, HIV-1 preferentially localized in a subset of small alveolar macrophages (14). Among CD4^+^ T cells, CCR4^+^CCR6^−^ T cells and CXCR3^+^CCR6^+^ T cells are highly permissive to both R5 and X4 HIV-1 viruses, whereas CXCR3^+^CCR6^−^ T cells are resistant to both R5 or X4 viruses (60).

Our data highlighted interesting differences between the non-adherent Siglec-1^hi^CD4^+^MDM and the adherent Siglec-1^Lo^CD4^−^MDM in their capacities to transfer HIV-1 to other MDM. Non-adherent Siglec-1^hi^CD4^+^MDM which were more restrictive to HIV-1 infection transferred virus to adherent Siglec-1^Lo^CD4^−^MDM. This suggests that Siglec-1^hi^CD4^+^MDM and Siglec-1^Lo^CD4^−^MDM may have distinct roles or functions in the context of HIV-1 infection. The restrictive phenotype of non-adherent Siglec-1^hi^CD4^+^MDM suggests that these cells primarily capture HIV-1 and could transfer the captured virus to other cells. In contrast, the permissive phenotype of the adherent Siglec-1^Lo^CD4^−^MDM suggests these as the preferential subset for HIV-1 infection. Indeed, the higher expression of integrins/adhesion molecules by the non-adherent Siglec-1^hi^CD4^+^MDM (LFA-1, ICAM-1, ICAM-2, ICAM-3, and ICAM-5) further suggest that they probably interact more efficiently with other cells compared to Siglec-1^Lo^CD4^−^MDM.

It has been previously demonstrated that differences in HIV-1 permissiveness has been related to the differential expression of intrinsic anti-HIV-1 cellular factors (61–63). We examined uninfected and HIV-1-infected Siglec-1^hi^CD4^+^MDM and Siglec-1^Lo^CD4^−^MDM, and compared the expression of several well-characterized cellular factors that restrict HIV-1 infection. The non-adherent Siglec-1^hi^CD4^+^MDM expressed higher levels of several restriction factor genes that affect HIV-1 replication and release of infectious virus, even prior to HIV-1 exposure. While some genes directly inhibit HIV-1, other genes may induce innate factors that restrict HIV-1 infection. The higher expression of TRIM22 (37, 64), BST2 (tetherin) (65–67), APOBEC (68–70), and SLFN11 (71–73), by non-adherent Siglec-1^hi^CD4^+^MDM indicated that these MDM may be more restrictive to HIV-1 infection. The higher expression of the cyclin-dependent kinases in Siglec-1^Lo^CD4^−^MDM may also explain the increased HIV-1 infectivity in these MDM despite their increased SAMHD1 expression profile. In addition, IL-12R1 and IL-12R2, IL-13, and IGF-1 genes were upregulated in the non-adherent Siglec-1^hi^CD4^+^MDM. Since these genes have been reported to inhibit viral replication, we speculate that they may contribute to the restrictive permissivity observed with the non-adherent Siglec-1^hi^CD4^+^MDM. Other methods including qPCR will be employed to confirm these data. This differential pattern of HIV-1 restrictive genes expression was maintained postinfection.

Our study identified two macrophage subsets that interact differentially with HIV-1. Siglec-1^hi^CD4^+^MDM, an HIV-1 restrictive subset, captures and transfers virus to macrophages. Siglec-1^Lo^CD4^−^MDM on the other hand, are highly permissive to HIV-1 infection, and may not readily transfer virus to macrophages. Should interventions be aimed at the permissive macrophage subset, or should it be focused on the restrictive macrophage subset that capture and facilitate transfer of virus to other cells? This highlights the complex role of macrophages in HIV-1 pathogenesis. Our data suggest that interventions should aim to block interactions between HIV-1 and macrophages.

## Ethics Statement

RV229B (WRAIR Protocol #1386): This protocol “Apheresis of blood components from healthy volunteers for *in vitro* research” and all related documents were approved by the following independent Institutional Review Boards: Division of Human Subject Protection, Walter Reed Army Institute of Research; Ethical Review Committee for Research in Human Subjects. All volunteers provided written informed consent following discussion and counseling by the clinical study team prior to enrollment and before the blood draw.

## Author Contributions

OJ designed the study. OJ, JK, ET, and SO performed the experiments. OJ, ET, and JK analyzed and interpreted the data. OJ wrote the manuscript. MR oversaw the experiments and edited the manuscript. OJ, MR, JK, ET, SO, CA, and NM reviewed the manuscript. All the authors have read the revised manuscript and agreed to submit it to Frontiers in Immunology.

## Disclaimer

This material has been reviewed by the Walter Reed Army Institute of Research. There is no objection to its publication. The opinions or assertions contained herein are the private views of the author and are not to be construed as official, or as reflecting true views of the Department of the Army or the Department of Defense.

## Conflict of Interest Statement

The authors declare that the research was conducted in the absence of any commercial or financial relationships that could be construed as a potential conflict of interest.
