# Supplementary figures and images for: Human Primary Macrophages Derived In Vitro from Circulating Monocytes Comprise Adherent and Non-Adherent Subsets with Differential Expression of Siglec-1 and CD4 and Permissiveness to HIV-1 Infection
Source: Front Immunol. 2017 Oct 23;8:1352. doi: 10.3389/fimmu.2017.01352 (PMC5662875; doi:10.3389/fimmu.2017.01352)

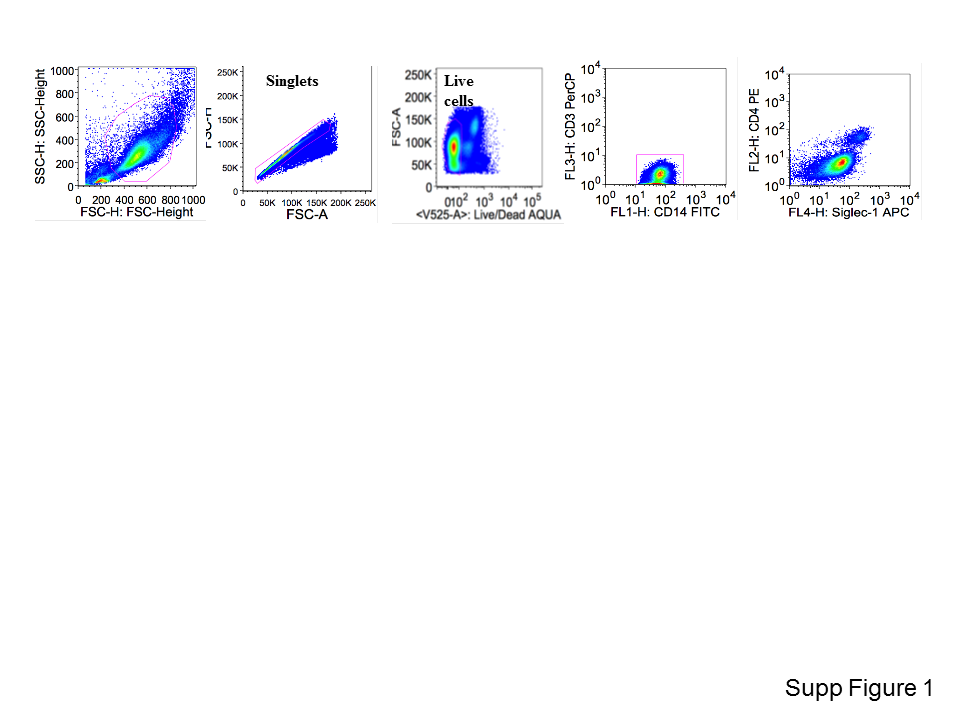

Supplement: Supplementary file 2 [file Image_1.TIF]

Genes CPM per Biotype

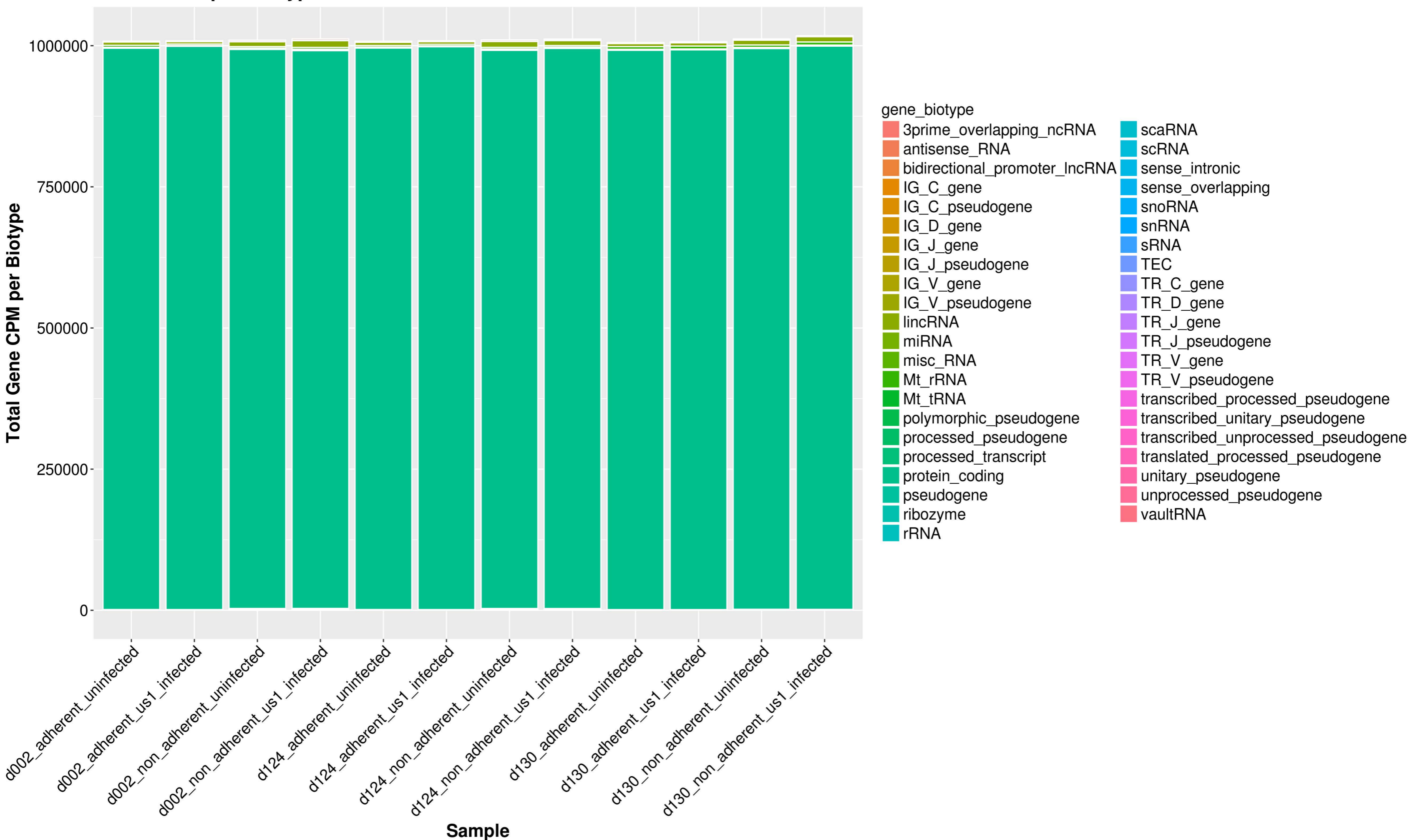

Supplement: Supplementary file 3 [file Image_2.PDF]

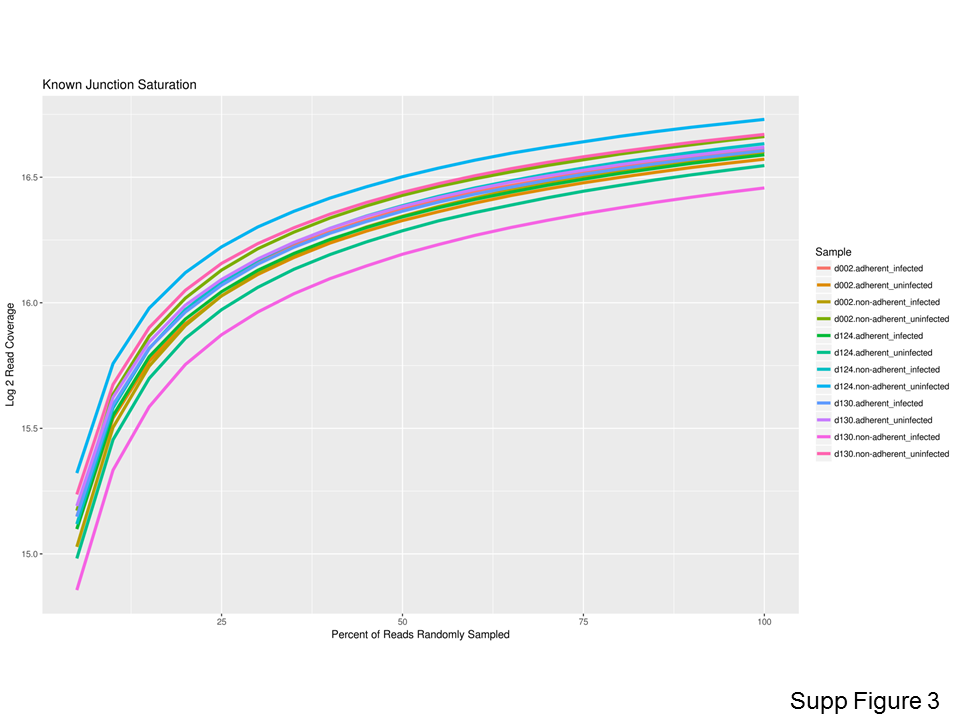

Supplement: Supplementary file 4 [file Image_3.TIF]

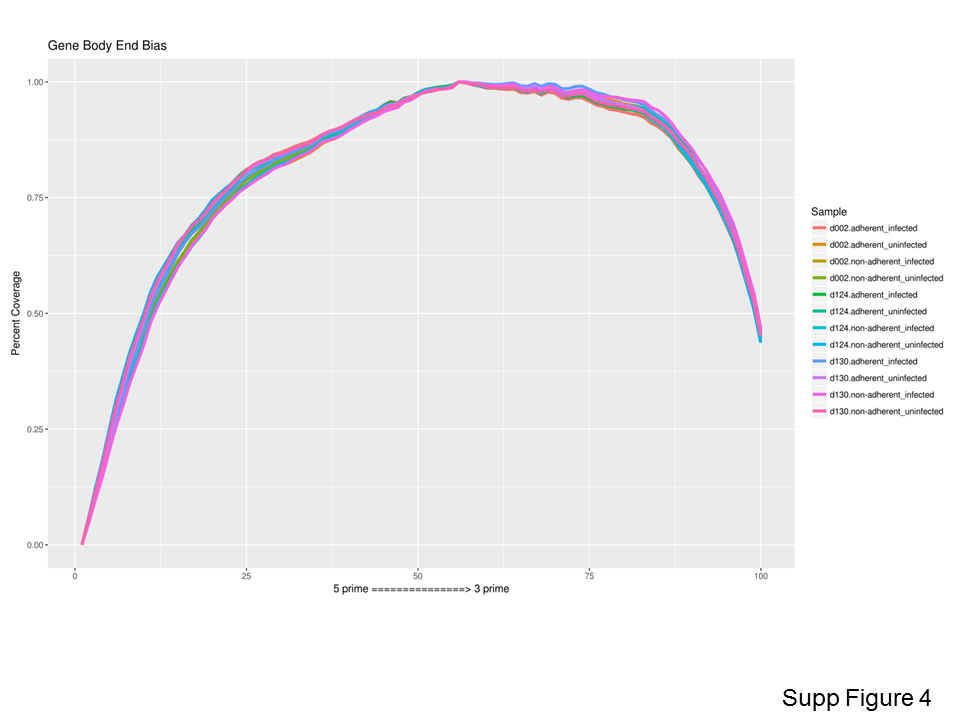

Supplement: Supplementary file 5 [file Image_4.TIF]

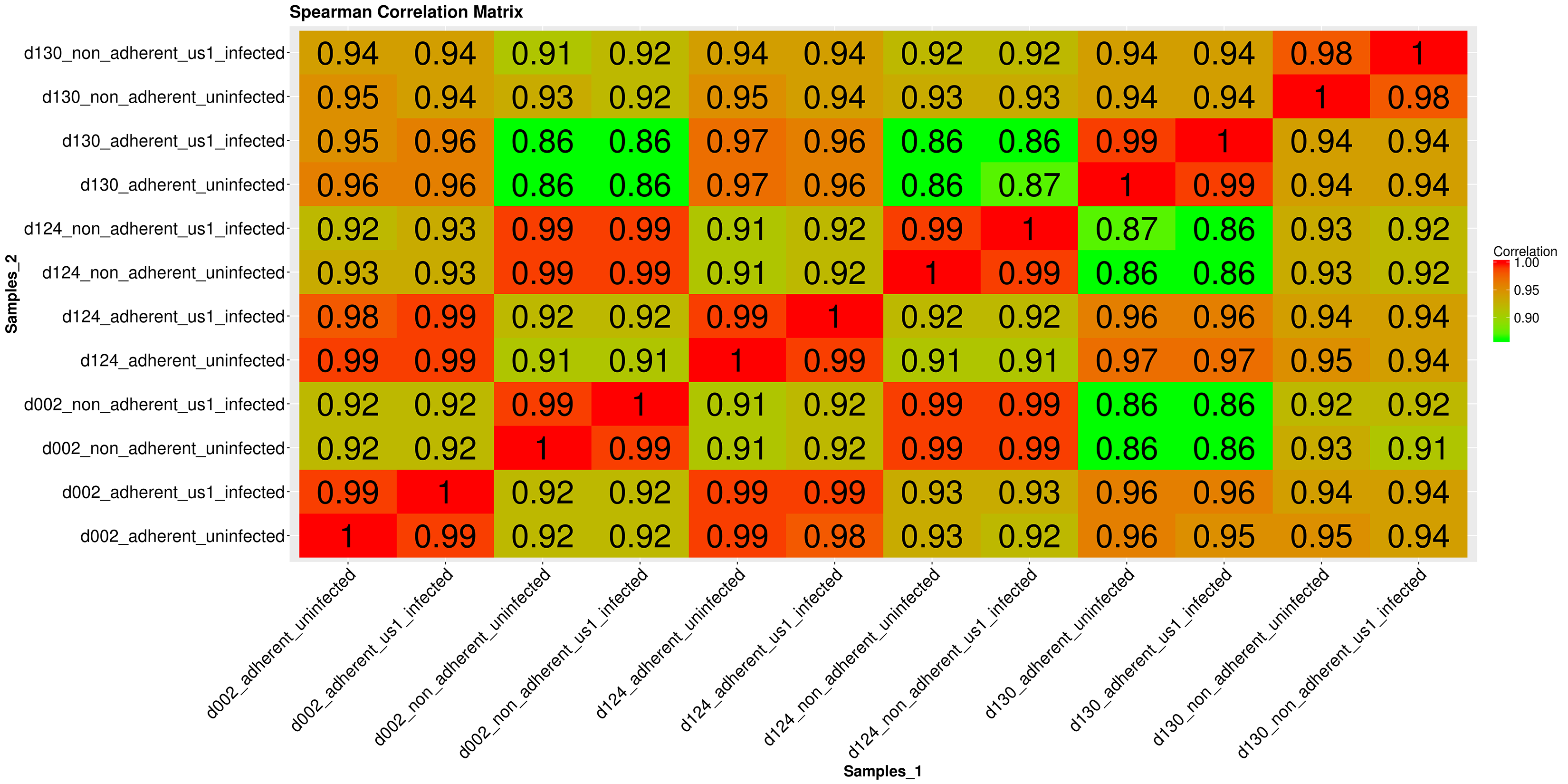

Supplement: Supplementary file 6 [file Image_5.TIF]

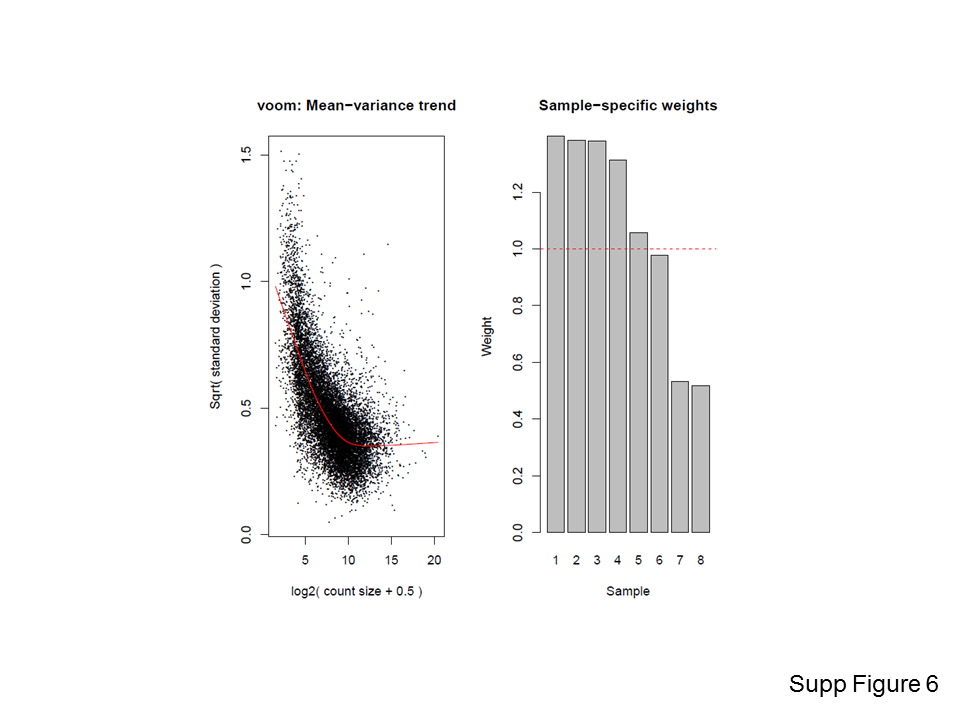

Supplement: Supplementary file 7 [file Image_6.TIF]
